# Pyo-septhamie in Foals Caused by Phlebitis Umbilicalis*Paper presented at the New York State Veterinary Medical Society.

**Published:** 1894-02

**Authors:** John Wende

**Affiliations:** Buffalo, N. Y.


### PYO-SEPTHAMIE IN FOALS CAUSED BY PHLEBITIS UMBILICALIS. *

By John Wende, V.S., Buffalo, N. Y.

The above appellation is used by Prof. Dickerhoff to designate the disease to which I wish to call your attention. Prof. Walley has given the term specific arthritis or synovitis; Prof. Williams describes the malady under the head of scrofulous ostitis, and considers it as scrofulous in its nature. Some writers view it as a constitutional disease, while others, especially the German veterinarians of to-day, accept it as pyæmic or septicæmic in origin, and a sequel to or identical with omphalo phlebitis. The disease has received attention from some of the leading veterinarians in Europe for the last one hundred years; Brugnone, of Turin, mentioned it as early as 1781. Dietrichs described the malady in the "Gestutskunde" in 1824, under the name of lahme, multiple arthritis, a disease of foals affecting the bones and joints, and about the same time Wirth designated the disease as acute epilepsie, and Hofacker confounded it with rachitis; Renard, a French veterinarian, reported the disease in 1828 existing as an enzootic at the large breeding establishments of France. In 1831 Strauss described the disease as a constitutional disturbance of the lymphatic system, especially the mesenteric glands. Traeger, in 1839, described it as due to a mal-condition of the blood. Then followed Heusinger, Funke, Spinola and Vieth, who were converts to the scrofulous hypothesis. Haubner, in 1840, being governed by its varied manifestations, described it as three different diseases affecting the suckling young, viz.: lahme, rheumatism and arthritis. The views of Hering in 1849 were that the malady was of constitutional origin, the nature of which hovered somewhere between rheumatism, tetanus and arthritis. This was followed by the theory of Furstenberg, as a fatty degeneration of the muscles, the cause of which he cast upon the great sheet anchor of catching cold. Then came Roloff with rachitis as the cause of the trouble. Thus the hypothetical confusion of this disease existed until 1873, when Bollinger, by holding repeated autopsies, discovered the true cause of this malady.

As you will infer from the term omphalo phlebitis, we have

---

*Paper presented at the New York State Veterinary Medical Society.

primarily an inflammation of the umbilicus and umbilical blood-vessels, which forms a distributing point and fountain-head for a metastatic pyæmia. The condition of the navel at or immediately after birth offers every opportunity for the absorption of a septic poison into the umbilical vein, there to regenerate itself a thousand fold in the thrombus remaining after the cessation of the fœtal circulation, from here the virus, in the form of small emboli, passes through the vena portal into the circulation, by which they are distributed to all parts of the body, lodging in the minute capillaries of any organ or tissue, manifesting its seat in due course of time by inflammation and suppuration, and according to the location of the embolism, you may have a varied number of complications, such as pneumonia, hepatitis, nephritis, cerebro spinal meningitis, arthritis, choroiditis, iritis, etc.

The symptoms vary according to localization and extent of complications, primarily you have hurried breathing, high fever, varying from 100 to 106 degrees F., utter want of appetite, distressed expression of countenance, patient usually preferring the recumbent position, manifesting great weakness if forced to rise, there may appear suddenly an intensely painful swelling of one or more joints, attributed generally to an injury incurred by the mare, these in turn may disappear as quickly as they developed, only to arise again in some other articulation with renewed virulence, usually terminating in suppuration. Moist and swollen condition of the navel, from the center of which there may be a discharge of ichorous pus, and immediately connected with this may be a pervious urachus, which is not, however, an imperative condition, and if present is readily recognized by the almost constant dribbling of urine from the umbilicus, which, during the first stages of the disease, is secreted in large quantities, light in color and acid in reaction, constipation of the bowels, which may soon be followed by diarrhœa, the fæcal matter being clay-colored and emitting an extremely offensive stench, fainting spells have been observed as the disease advances, abscesses appear in various parts of the body, and if not opened surgically will burst, allowing the escape of a creamy and offensive smelling pus, the discharge being especially fœtid if joints and ligaments are affected.

Post-mortem examinations of colts that have died with this malady, disclose many lesions, such as dilatation of the umbilical vein containing a thrombus surrounded by an accumulation of discolored or greyish-white purulent matter, deep seated muscular and superficial abscesses, liver is enlarged, friable and clay-colored, with localized accumulations of pus, inflammation of the lungs, with abscesses in their parenchyma, the latter may open into the bronchial tubes, thereby producing a diffuse purulent bronchitis, pleuritis, with serous exudate in thoracic cavity. The heart, spleen and kidney present structural changes, inflammation of the bowels extending to the mesenteric glands, arthritis with purulent infiltration, often to such an extent that bones, cartilages, ligaments and periosteum have become macerated.

If the veterinarian is called in during the first stages, when the disease has not extended beyond the navel and navel vein, then a favorable prognosis can be given, as the omphalitis is only local and amenable to treatment. If, on the other hand, he is not consulted until a generalized metastatic pyæmia has been established, then the prognosis must be guarded. Siedamgrotzky places the death rate at seventy-five per cent. Hering cites a record kept at a breeding establishment in Germany for fifteen years, and says that of one hundred and eighty-seven colts which died there during the interval, eighty five succumbed to this malady, and of sixty-seven colts attacked by the disease, forty-seven died before reaching the age of three weeks.

In order to prevent the trouble the mare should be placed in a stall before foaling, where the surroundings are in a perfect hygienic condition, and not into a stall that has been used for the purpose recently, unless it has been thoroughly cleansed and disinfected, if the season of the year and other conditions are favorable, a clean pasture field should be preferred. If the colt is foaled in pasture, let it alone by all means, unless there are indications of disease. If born in stable, have the latter kept clean, wash the navel several times daily with some antiseptic lotion, or dust the parts with equal portions of iodoform and boracic acid, over which apply a pledget of absorbent cotton, to be kept in place by a bandage around the body. This should be carefully attended to until all danger of infection at the umbilicus is past. Ligating the latter as recommended by some authors should not be practiced under any circumstances. Some German authors advise an application of pine tar to the navel at, or soon after, birth.

If the disease is once established, treatment cannot be recommended too soon. If, in the first stages, the patient should be laid on its back and held by attendants, the umbilical region should be thoroughly washed with some antiseptic solution, then grasp the navel with one hand, with a sharp scalpel in the other make an incision long and deep enough to find the affected vein, which then should be thoroughly washed out with a ten per cent. solution of creolin, using for the operation a medium-sized human catheter connected to a rubber bulb syringe. Do this several times daily ; dust the parts with an equal mixture of iodoform and boracic acid, over which apply a pledget of absorbent cotton, to be kept in place by a bandage around the body. If abscesses have formed they should be opened at once, unless located in the joints, then should be aspirated, and the cavity washed out with a saturated solution of boric acid, other abscesses to receive the same attention as the umbilicus. Internal remedies should be such as will counteract the conditions present in the case. If constipation manifests itself, in summer give small doses of Epsom salts, in cold weather sweet oil in doses from one to two ounces is preferable, until the bowels regain their normal condition. If diarrhœa exists this should be overcome with vegetable tonics and astringents, in extreme cases bismuth sub. nitras may be given in ten to twenty grain doses two or three times daily. If the circulation is weak and requires stimulation, give spirits of camphor, one to two drachms diluted in milk. Salicylate of soda and quinine can also be given in their respective doses, but experience has taught me to give the colt as few internal remedies as possible, and at the same time to feed the dam a generous diet in which is mixed large doses of hyposulphite of soda and powdered gentian root.

If pervious urachus exists, inject the ordinary white lotion, or a solution of nitrate of silver, five grains to the ounce of water several times daily.

